# Chloral, the New Sedative and Anæsthetic

**Published:** 1869-08

**Authors:** 


### Chloral, the New Sedative and Anæsthetic.

This is one of the last novelties produced in the Berlin medical world. Dr. Liebrich, its discoverer, reminds us that it evolves chloroform when treated with an alkali, and expects to produce the effects of chloroform by injecting it into the (alkaline) blood.

It produces sound, death-like sleep, not followed by nausea. Rabbits partake immediately and freely of food after waking from the chloral-sleep.

In one case, four grains were taken internally, and two grains injected subcutaneously; the patient slept fourteen hours, and wakened without nausea or headache, and took food rapidly.—*Medical Gazette.*

